# Transplanting neurofibromatosis-1 gene knockout neural stem cells improve functional recovery in rats with spinal cord injury by enhancing the mTORC2 pathway

**DOI:** 10.1038/s12276-022-00850-9

**Published:** 2022-10-14

**Authors:** Guoliang Chen, Xianlong Li, Hongzhang Zhu, Huachuan Wu, Dacheng He, Liangyu Shi, Fuxin Wei, Xizhe Liu, Ningning Chen, Shaoyu Liu

**Affiliations:** 1 Guangdong Provincial Biomedical Innovation Platform of Regeneration and Repair of Spinal Cord and Nerve Injury, Department of Orthopedic Surgery, The Seventh Affiliated Hospital, Sun Yat-sen University, No. 628 Zhenyuan Road, Shenzhen, 518107 China; 2 Guangdong Provincial Key Laboratory of Orthopaedics and Traumatology /Orthopaedic Research Institute, Department of Spine Surgery, The First Affiliated Hospital, Sun Yat-sen University, No. 58 Zhongshan 2nd Road, Guangzhou, 510080 China; 3 Department of Radiology, The First Affiliated Hospital, Sun Yat-sen University, No. 58 Zhongshan 2nd Road, Guangzhou, 510080 China

**Keywords:** Neural stem cells, Neural stem cells

## Abstract

The poor survival and low efficiency of neuronal differentiation limits the therapeutic effects of transplanted neural stem cells in the treatment of spinal cord injury. Neurofibromatosis-1 (*NF-1*) is a tumor suppressor gene that restricts the rapid and abnormal growth and differentiation of neural cells. In the present study, lentiviral vectors were used to knock out *NF-1, Ricotr* (the core member of mTORC2) or *NF-1*+*Ricotr* in neural stem cells in vitro, and the *NF-1*, *Ricotr* or *NF-1*+*Ricotr* knockout neural stem cells were transplanted at the lesion site in a rat model of spinal cord injury (SCI). We first demonstrated that targeted knockout of *NF-1* had an antiapoptotic effect and improved neuronal differentiation by enhancing the mTORC2/Rictor pathway of neural stem cells in vitro. Subsequently, transplanting *NF-1* knockout neural stem cells into the injured site sufficiently promoted the tissue repair and functional recovery of rats with spinal cord injury by enhancing the survival and neuronal differentiation of grafted neural stem cells. Collectively, these findings reveal a prominent role of *NF-1* in neural stem cell biology, which is an invaluable step forward in enhancing the benefit of neural stem cell-mediated regenerative cell therapy for spinal cord injury and identifies the transplantation of *NF-1* knockout neural stem cells as a promising strategy for spinal cord injury.

## Spinal cord injury: NF-1 knockout improves stem cell transplant survival

Knocking out a tumor-suppressing gene improves the success of stem cell transplants for spinal cord injury. A spinal cord injury destroys neural tissues and creates an environment where it is difficult for neurons to regenerate. This has initiated a hunt for robust stem cells that could survive transplants into damaged areas. Ningning Chen at Sun Yat-sen University in Shenzhen, China, and co-workers hypthesized that a tumor suppressor gene called *NF-1* may restrict the rapid growth and differentiation of transplanted neural stem cells. They found that neural stem cells with *NF-1* knocked out survived for longer and differentiated more effectively into neurons, thanks to a signaling pathway that *NF-1* normally suppresses. The cells also promoted tissue repair and functional recovery when transplanted into injured sites in rats, indicating a promising therapeutic strategy.

## Introduction

Spinal cord injury (SCI) is one of the most serious traumatic neurological conditions worldwide, owing to the mostly irreversible detrimental outcomes^1^. The major pathological changes of SCI include neural and microvasculature structural damage directly caused by a primary acute phase followed by secondary damage^2,3^. These pathological processes not only directly destroy the neural tissues but also inhibit neural regeneration due to the undesirable postinjury microenvironment^4^. Therefore, recruiting newborn neurons to the injury site might be effective for promoting neurogenesis and functional improvement following SCI^5^. Several studies have demonstrated that endogenous nerve regeneration is not sufficient after SCI^6^. Moreover, neural stem cells (NSCs) are multipotent, self-renewing stem cells and can differentiate into specific neural cells^7^. Thus, NSC transplantation has been considered a promising therapy for SCI to replace lost tissues and secrete anti-inflammatory factors to improve the hostile microenvironment^8^. However, a multitude of studies have revealed that the unfavorable SCI microenvironment leads to the limited survival of transplanted NSCs, and a large proportion of transplanted NSCs differentiate into astrocytes rather than neurons to form a glial scar^9,10^. To prevent this poor survival and differentiation and to yield more functional neurons, researchers have conducted genetic modifications and preconditioning of transplanted NSCs^11^. Recently, the roles of the neurofibromatosis-1 (NF-1) tumor suppressor gene (*NF-1*, encodes neurofibromin) in the development and differentiation of the central nervous system have attracted attention^12–15^. As a tumor suppressor gene, *NF-1* may restrict the rapid and abnormal growth and differentiation of neural cells. The genetic modification of *NF-1* was proven to affect the fates of NSCs. Several studies have reported the trilineage potential of NSCs revealed by *NF-1* inactivation. Chen et al. showed that *NF-1* regulated NSC proliferation and multilineage differentiation through distinct RAS effector pathways^12^. Furthermore, the knockout of *NF-1* was demonstrated to unlock a latent oligodendrocyte lineage potential to produce all three lineages from NSCs in vivo^14^. Moreover, Dahiya et al. demonstrated that *NF-1* inactivation led to increased NSC proliferation and gliogenesis in the optic chiasm and brainstem but not in the cerebral cortex^13^, which may be attributed to the differential Rictor expression in the different brain regions, leading to region-specific mTOR/Rictor-mediated Akt phosphorylation and Akt-regulated p27 phosphorylation^16^. Current studies related to *NF-1* and NSC differentiation are confined to brain NSCs, and evidence indicates differences in NSC biology in different brain regions^13,15,16^. However, it remains unclear whether *NF-1* plays a role in the cell biology of transplanted NSCs in SCI. One recent work suggested that the activation of mTORC2 contributes to the intrinsic axon growth capacity in adult sensory neurons after injury^17^. Moreover, localized Rictor overexpression is considered a promising potential strategy for SCI because it facilitates neurogenesis^18^. Based on these findings, we speculated that *NF-1* might regulate the fates of transplanted NSCs via the mTORC2 signaling pathway in SCI.

In this study, we employed a lentiviral vector carrying the CRISPR/CAS9 system to transfect rat NSCs for the targeted knockout of *NF-1* and *Rictor*. Then, we injected the transfected NSCs into a rat SCI model to investigate the effects of *NF-1* knockout on NSC survival and differentiation and the potential mechanisms underlying these effects. Our findings first demonstrate that knocking out *NF-1* increases the number of NSCs at the injury site and promotes transplanted NSCs to differentiate into neurons for neurogenesis. Importantly, knockout of *NF-1* attenuated glial scar formation and dramatically improved locomotor function in injured rats. Signaling pathway analysis by Western blot analysis revealed that *NF-1* regulates NSC differentiation associated with the mTORC2 signaling pathway (Supplementary Fig. 1, Graphical abstract). Taken together, our study provides molecular insights into the *NF-1*-mediated neuronal differentiation of transplanted NSCs and identifies the transplantation of *NF-1* knockout NSCs as a potential therapy for SCI.

## Materials and methods

### Ethical approval

All procedures performed on experimental animals were approved by The Seventh Affiliated Hospital of Sun Yat-sen University Ethics Committee (2019SYSUSHDW-013) and implemented according to the guidelines established by the Ministry of Health of China.

### The construction of lentivirus

The lentiviral vectors (LV) carrying the CRISPR/CAS9 system and a sequence that knocked out *NF-1* or *Rictor* were constructed by GeneChem Company (Shanghai, China) and labeled with green fluorescent protein (GFP). The nucleotide sequences of vector, *NF-1*, and *Rictor* used in the construction of lentivirus were as follows:

vector: 5′-CGCTTCCGCGGCCCGTTCAA-3′

NF-1: 5′- TCCGAAGTTCGGCTGCATGT -3′

Rictor: 5′- ATAGCGCAGCGCTCGCAACC -3′

### NSC isolation and culture

NSCs were obtained from the fetal brains of embryonic Day 14 pregnant Sprague‒Dawley rats (Experimental Animal Center of Southern Medical University, Guangzhou, China) according to our previous protocol^19^. In brief, the brain tissues of rat embryos were mechanically dissected and dissociated in Hanks’ balanced salt solution and then filtered twice with filters with a 40 μm aperture. The cell suspension was centrifuged at 1000 rpm for 5 min. The supernatant was discarded, and the cell pellet was resuspended to a single cell suspension. NSCs were seeded on T25 culture flasks with expansion medium containing Dulbecco’s modified Eagle’s medium/F-12 nutrient mixture, 2% B-27 supplement, 1% penicillin/streptomycin, 1% L-glutamine, 20 ng/mL fibroblast growth factor-2 (FGF-2) and 20 ng/mL epidermal growth factor (EGF). NSCs were cultured at 37 °C in 5% CO_2_ and passaged via digestion with Accutase when the neurosphere diameter was close to 100 μm. All NSCs used in this study were in passages 2–3.

### NSC transfection and induced neuronal differentiation

For cell transfection, the neurospheres were dissociated into a single cell suspension and plated on coverslips coated with 0.01% poly-L-lysine at a density of 5 × 10^4^ cells/well in 12-well plates or a density of 1 × 10^6^ cells/well in 6-well plates. Cells were maintained in the expansion medium for 24 h to recover from the passage. After recovery, the cells were transfected with the control lentivirus (LV-vector), NF-1 sgRNA-CAS9 lentivirus (LV-NF-1), Rictor sgRNA-CAS9 lentivirus (LV-Rictor), and NF-1 sgRNA-CAS9 lentivirus + Rictor sgRNA-CAS9 lentivirus (LV-NF-1+LV-Rictor), with a multiplicity of infection of 10. Cells were seeded and cultured as usual in the control group. Twenty-four hours later, the expansion medium was changed to differentiation medium containing Neurobasal Medium, 2% B-27 supplement, 1% penicillin/streptomycin, and 1% L-glutamine. GFP expression was visualized by fluorescence microscopy (DM6B, Leica, Germany) after 48 h of transfection. The cell viability and transfection efficiency were evaluated by counting the number of GFP-positive cells from five random fields among all cells. Cells were harvested for immunofluorescent staining and Western blot analysis 14 days after inducing differentiation.

### Induced apoptosis of NSCs with tumor necrosis factor-alpha (TNF-α)

To analyze the antiapoptotic ability of *NF-1*, we conducted apoptosis induction according to our previous study^20^. Briefly, 24 h after transfection, the primary medium was changed, and the cells were cultured in expansion medium for another 48 h. Then, the primary medium was removed, and fresh expansion medium containing 50 ng/ml TNF-α was added to the NSCs and incubated at 37 °C and 5% CO_2_. Twenty-four hours later, the cells were collected for further assessments by terminal deoxynucleotidyl transferase-mediated dUTP nick-end labeling (TUNEL) assays and Western blot analysis.

The materials and reagents used for the cell experiments are listed in Table 1.

**Table 1:** The reagents and materials used in cell experiments.

Reagents and materials	Manufacturer
Dulbecco’s modified Eagle’s medium/F-12 nutrient mixture	Gibco, Grand Island, USA
B-27 supplement	Gibco, Grand Island, USA
Penicillin/streptomycin	Gibco, Grand Island, USA
L-glutamine	Gibco, Grand Island, USA
Fibroblast growth factor-2	Peprotech, Rocky Hill, USA
Epidermal growth factor	Peprotech, Rocky Hill, USA
Accutase	Thermo Fisher Scientific, Waltham, USA
Poly-L-lysine	Sigma, Germany
Neurobasal medium	Thermo Fisher Scientific, Waltham, USA
Tumor necrosis factor-alpha (TNF-α)	Peprotech, Rocky Hill, USA
T25 culture flask	Corning, Acton, USA
12-well plate	Corning, Acton, USA
6-well plate	Corning, Acton, USA
Coverslips	Thermo Fisher Scientific, Waltham, USA

### Surgical procedures and cell transplantation

A total of 130 adult female Sprague‒Dawley rats (weighing 200–220 g, supplied by the Experimental Animal Center of Sun Yat-sen University, Guangzhou, China) were divided into six groups according to the different treatments: sham (*n* = 25), control (*n* = 25), SCI+LV-vector NSCs (*n* = 25), SCI+LV-NF-1 NSCs (*n* = 25), SCI+LV-Rictor NSCs (*n* = 15) and SCI+LV-NF-1+LV-Rictor NSCs (*n* = 15). After 72 h of lentiviral transfection, NSCs were collected for transplantation. Briefly, after a week of adaptive feeding, buprenorphine (0.05 mg/kg) was subcutaneously administered to each rat 30 min before surgery. Deep anesthesia was induced with a 5% mixture of isoflurane in the air followed by intraperitoneal injection of ketamine (75 mg/kg) and xylazine (10 mg/kg). Then, the spinal cord was exposed at the 10th thoracic vertebral level (T10) via laminectomy. The animals in the sham group underwent spinal cord exposure but no injury. The rats in the control group received complete spinal cord transection but no NSC transplantation. In the other four groups, the exposed spinal cord was cut off using a microscissor for complete transection. Once the bleeding was stopped, the rats were implanted with 5 μL of LV-vector-, LV-NF-1-, LV-Rictor-, and LV-NF-1+LV-Rictor-transfected NSCs rostral and caudal to the injured site at a depth of 1 mm using a 5 μL Hamilton syringe with a 32G needle at an injection rate of 1 μL/min. The needle was left in the injected site for 3 min to allow cell diffusion and prevent leakage or backflow. The number of transplanted NSCs for each rat was 1 × 10^6^^19^. The muscle and the skin were sutured using 5–0 sutures in layers. All procedures were completed by the same group of researchers who were experienced in rat SCI modeling. After surgery, 1 mL of saline containing 1 × 10^5^ units of penicillin and 1 mg/kg meloxicam was intraperitoneally injected daily for 1 week to protect against infection and dehydration and to alleviate pain. The bladder of the rats was manually expressed twice daily until urinary function was restored. Rats were kept in the specific pathogen-free laboratory with a 12 h light/12 h dark cycle and conventional feeding until eight weeks after surgery.

### Functional evaluation

The 22-point (0–21) Basso, Beattie, and Bresnahan (BBB) open-field locomotor test was used to assess hindlimb locomotor function, including joint movements, stepping ability, coordination, and trunk stability^21^. The duration of each session was 5 min per rat. Finally, an overall score was calculated. The evaluation was independently performed by the same two observers who were blinded to the treatment of the tested animals and repeated three times.

### Magnetic resonance imaging (MRI)

MRI was performed using a 3.0 Tesla Siemens Verio MR scanner equipped with high-performance gradient coils eight weeks postinjury. A special eight-channel wrist coil was used to image the rats. Five animals were observed for each group. For scanning, the rats were subjected to general anesthesia, and each rat was subsequently placed in the ventral recumbent position using a surface coil for spinal cord measurements. T1- and T2-weighted MRIs of the spinal region were reconstructed in the sagittal plane to visualize the extent of the lesion. The cavity volume of the injured spinal cord was evaluated by contrasting the normal T8–10 spinal cord diameter in the midsagittal slides^19,22^.

### Spinal cord–evoked potential (SCEP) recording

Rats were anesthetized and stereotaxically fixed. The T5–T6 and T12–L1 vertebrae were completely exposed. Briefly, the stimulation electrode was inserted into the T5–T6 interspinous ligaments, and a pair of needle electrodes were inserted into the interspinous ligaments of T12–L1 for SCEP recording. Then, the electrodes were connected to a BL-410E Data Acquisition Analysis System for Life Science (Chengdu, China). The variables of the SCEP signals were set according to previous reports as follows: gain of 2000 time constant of 0.01 s and filtering at 300 Hz. For an SCEP, a single pulse stimulation (50 ms in duration at a frequency of 5.1 Hz and a voltage increase of 1 mV) was transmitted through the electrodes until a mild twitch of the vertebral body of the animal was observed. One hundred SCEP responses were averaged for each rat to obtain high-quality waveforms for the SCEP signals.

### Tissue processing

At eight weeks after SCI, rats were deeply anesthetized and transcardially perfused with 500 mL of 0.9% normal saline. Then, 1 cm samples of spinal cord tissue containing the lesion center were promptly extracted from the animals (*n* = 5 per group) and used for Western blot analysis. Other rats continued to be perfused with 300 mL of phosphate-buffered saline (PBS; pH 7.4) followed by 4% paraformaldehyde (PFA). The T8–T11 cord segments were dissected and then postfixed overnight in 4% PFA and soaked at 4 °C overnight in 10% sucrose, followed by 30% sucrose until the tissue sank to the bottom. The specimens were embedded in an optimal cutting temperature (OCT) compound, frozen at –20 °C, and sliced at a thickness of 10 μm in the sagittal or transverse plane by a cryostat (Thermo Fisher Scientific, Waltham, USA).

### Immunofluorescence staining

Coverslips with cells (*n* = 5 per group) or tissue sections of the spinal cord (*n* = 5 per group) were fixed in 4% PFA for 30 min and then permeabilized and blocked with 0.3% Triton X-100 combined with 5% BSA for 1 h. The coverslips with cells or tissue sections were incubated overnight at 4 °C in primary solution at a 1:300 dilution. After three washes in PBS, the primary antibodies were probed with the secondary antibodies at a 1:500 dilution for 1 h at room temperature. Finally, the coverslips or tissue sections were washed in PBS three times and mounted using Prolong Gold Antifade Reagent containing 4′-6-diamidino-2-phenylindole (DAPI) (Invitrogen, Carlsbad, USA). The targeted marker-positive cells in each visual field were counted under a confocal laser scanning microscope (880, Carl Zeiss AG, Germany). Five random fields in each coverslip or tissue section from each group were examined. The antibodies used are listed in Table 2.

**Table 2:** The antibodies used in immunofluorescence staining (IF) and Western blot (WB).

Name	Primary/secondary	Application	Manufacturer
Anti-Nestin antibody	primary	IF	CST (Danvers, USA)
Anti-SOX2 antibody	primary	IF	CST
Anti-MAP2 antibody	primary	IF and WB	CST
Anti-β3-Tubulin antibody	primary	IF and WB	CST
Anti-GFAP antibody	primary	IF and WB	CST
Anti-NF-200 antibody	primary	IF and WB	CST
Anti-NeuN antibody	primary	IF and WB	CST
Anti-cleaved caspase-3 antibody	primary	IF and WB	CST
Anti-neurofibromin antibody	primary	WB	CST
Anti-P-mTOR antibody	primary	WB	CST
Anti-Rictor antibody	primary	WB	CST
Anti-GAPDH antibody	primary	WB	CST
Anti-Bcl-2 antibody	primary	IF and WB	CST
Anti-CNPase antibody	primary	IF and WB	CST
Anti-myelin basic protein (MBP) antibody	primary	IF and WB	CST
Alexa Fluor 594 goat anti-rabbit antibody	secondary	IF	Invitrogen (Carlsbad, USA)
Alexa Fluor 594 goat anti-mouse antibody	secondary	IF	Invitrogen
Goat anti-rabbit antibody	secondary	WB	Invitrogen
Goat anti-mouse antibody	secondary	WB	Invitrogen

*CST* Cell Signaling Technology, *IF* immunofluorescence staining, *WB* Western blot.

### Western blot

Protein lysates were extracted from the cells plated on six-well plates (*n* = 5 per group) at 14 days after transfection and from the tissue of spinal cord injury. Cells and tissues were lysed in RIPA buffer, and total protein was extracted. Then, the protein concentration was determined using a bicinchoninic acid assay (Beyotime Biotechnology, Shanghai, China) and equalized before loading. Protein samples were separated by SDS‒PAGE (10% Bis-Tris Gel), transferred to polyvinylidene fluoride membranes (Millipore, Bedford, USA), and blocked with 5% nonfat dry milk (Sigma, Germany) in TBST (50 mM Tris, 150 mM NaCl, 0.1% Tween 20, pH 7.6) for 1 h at room temperature followed by incubation with primary antibodies at a 1:1000 dilution at 4 °C overnight. After three washes in TBST, the PVDF membranes were incubated with peroxidase-conjugated secondary antibody for 1 h at room temperature and washed in TBST three times. Immunolabeling was detected using an enhanced chemiluminescence system (Millipore, Bedford, USA). The antibodies used are listed in Table 2.

### TUNEL assay

The TUNEL assay was conducted by using the TUNEL kit (Roche, Reinach, Switzerland). After 30 min of fixation with 4 % PFA and 15 min of permeabilization with 0.3 % Triton X-100, the cells were incubated with TUNEL reagent for 1 h at 37 °C in the dark. Finally, stained cells were analyzed under a confocal laser scanning microscope.

### Axonal tracing

In the 7th postoperative week, the rats (*n* = 5 per group) were randomly selected for retrograde labeling of host axonal tracts. The cell bodies of neurons projecting to the spinal cord caudal to the transplanted area were labeled with Fluorogold (FG; Biotium, San Francisco, USA). Briefly, a dorsal laminectomy was performed at T12, and 0.5 μl of FG was injected into the spinal cord using a Hamilton syringe. At 1 week after injection, the animals were sacrificed, and the T8 segment of the spinal cord was removed, cryopreserved in graded sucrose solutions, and sliced into 10 μm frozen sections. The sections were observed via a confocal laser scanning microscope (880, Carl Zeiss AG, Germany) to detect and count FG-labeled neurons in the ventricles. The number of FG-labeled axons was assessed by two independent examiners who were blinded to the rats’ treatment status. The rats injected with FG were not used for functional evaluation.

### Hematoxylin-eosin (HE) staining

For visualization of the cavity area, samples (*n* = 5 per group) were used for HE staining. The T8–T11 longitudinal spinal cord sections from each group were stained with HE according to standard protocols and observed under a brightfield microscope (BX53, Olympus, Japan).

### Myelination observation

For myelination assessment, rats (*n* = 5 per group) were anesthetized at 8 weeks after SCI and perfused with 2.5% glutaraldehyde (Kemiou, Tianjin, China; 25% glutaraldehyde: 4% PFA = 1:9). The harvested tissues were postfixed in 2.5% glutaraldehyde, subsequently incubated in 1% osmium tetroxide, and then cleared in propylene oxide post-dehydration in a series of graded alcohols. After being embedded in pure Epon, transverse semithin section (1 μm) were obtained and stained with 1% toluidine blue. For electron microscopy (EM), 50 nm ultrathin sections were required and double-stained with 2% uranyl acetate and led citrate for EM analysis. The number of myelinated axons was counted, and the morphology of myelin was observed under EM.

### Nissl staining

Nissl staining (*n* = 5 per group) was performed on transverse sections rostral to the epicenter with 0.1% cresyl violet (Sigma‒Aldrich, C5042) for 20 min at 37 °C. After the samples were rinsed in distilled water, the stained sections were differentiated in 95% ethyl alcohol. Then, the sections were dehydrated in increasing concentrations of ethyl alcohol and cleared in xylene. Subsequently, these sections were imaged and quantified under an optical microscope (BX53, Olympus, Japan) with high magnification. A total of five sections were quantified for each rat, and the average number of each group was calculated to determine neuronal survival.

### Statistical analysis

All statistical analyses were performed using GraphPad Prism 8 software (GraphPad Software, Inc., USA). All data in the study were first tested for normality using the Shapiro–Wilk test, which revealed a normal distribution. All data are expressed as the means ± SDs and were analyzed via one-way ANOVA followed by Bonferroni post hoc tests for multiple comparisons or Student’s t test for pairwise comparisons. *P* < 0.05 was considered to be statistically significant.

## Results

### Knockout of *NF-1* enhanced the antiapoptotic ability of NSCs in vitro

**Fig. 1:**
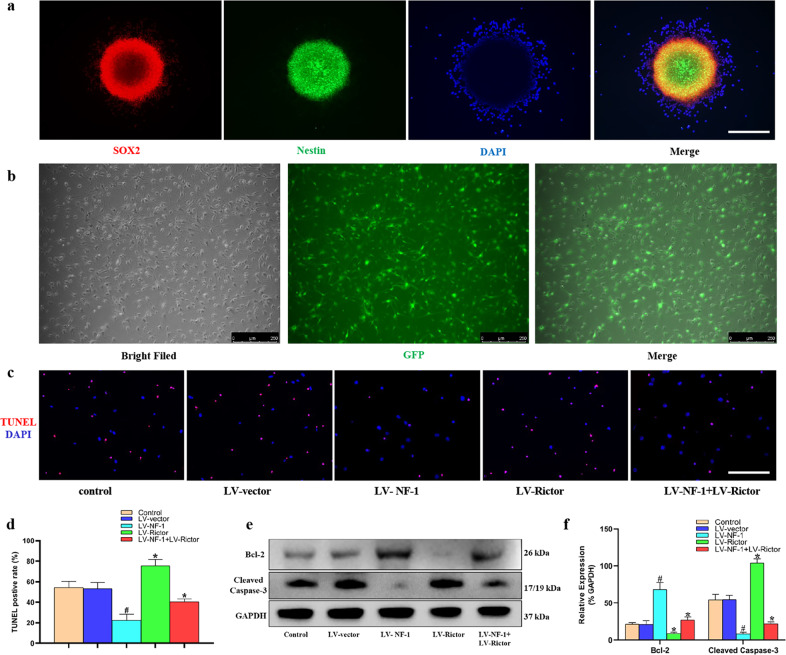
Knockout of *NF-1* improved the antiapoptotic ability of neural stem cells (NSCs) in vitro. **a** NSCs gathered as neurospheres and abundantly expressed SOX2 and Nestin (scale bar = 100 µm). **b** After successful transfection by lentivirus, most of the NSCs expressed green fluorescent protein (GFP) (scale bar = 250 µm). **c** NSCs after inducing apoptosis were detected through terminal deoxynucleotidyl transferase-mediated dUTP nick-end labeling (TUNEL) (*red*: TUNEL-positive; *blue*: 4′-6-diamidino-2-phenylindole, DAPI) (scale bar = 100 µm). **d** Quantitative comparison of TUNEL-positive cells in different groups. **e** Western blotting of apoptosis-related proteins, including Bcl-2, cleaved caspase-3, and glyceraldehyde-3-phosphate dehydrogenase (GAPDH). **f** Quantitative comparison of the expression of Bcl-2 and cleaved caspase-3 in different groups (data are expressed relative to GAPDH). *P < 0.05 compared with the control and LV-vector groups; ^#^*P* < 0.05 compared with the LV-NF-1+LV-Rictor group.

The NSCs gathered as neurospheres and abundantly expressed SOX2 and Nestin (Fig. 1a), which confirmed that the cells used in this study were NSCs. After transfection with the lentivirus, most of the NSCs expressed GFP (Fig. 1b). These data suggest successful transfection.

After successful lentivirus transfection, NSCs were treated with TNF-α. TUNEL staining showed that compared with that of the control and LV-vector-treated NSCs, the number of TUNEL-positive cells significantly decreased in the *NF-1* knockout NSCs but increased overwhelmingly in the *Rictor* knockout NSCs. The proportion of TUNEL-positive cells in the LV-NF-1+LV-Rictor treated NSCs was between those of the *NF-1* knockout and *Rictor* knockout NSCs (Fig. 1c, d). Similar expression of cleaved caspase-3 and Bcl-2 was detected by Western blots (Fig. 1e, f).

### Knockout of *NF-1* promoted neuronal differentiation and inhibited astrocyte differentiation of NSCs by enhancing the mTORC2 pathway in vitro

**Fig. 2:**
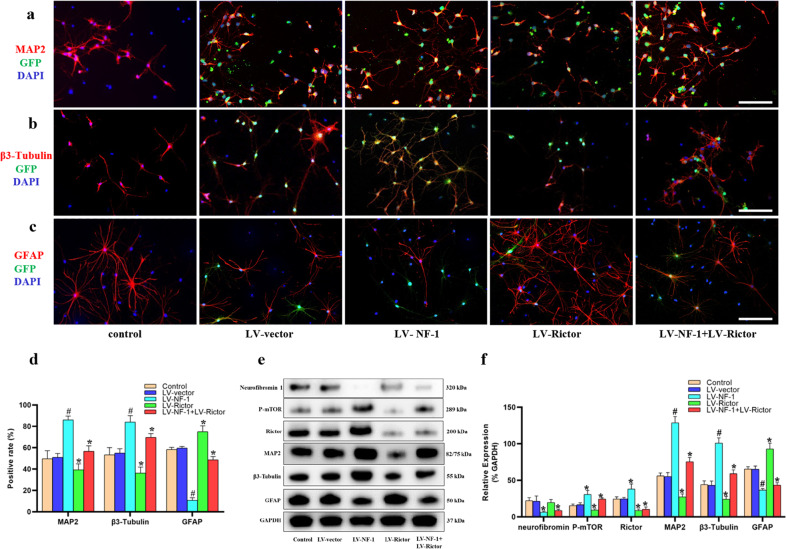
Knockout of *NF-1* promoted neuronal differentiation of neural stem cells (NSCs) by enhancing the mTORC2 pathway in vitro. **a**–**c** Immunofluorescence staining of neuronal markers (β3-Tubulin and MAP2), an astrocyte marker (GFAP), and green fluorescent protein (GFP) (scale bar = 100 µm). **d** Quantitative comparisons (positive rates) of β3-Tubulin-, MAP2- and GFAP-positive cells in different groups. **e** Western blotting of mammalian target of rapamycin complex 2 (mTORC2) signal pathway-related proteins (neurofibromin, phosphorylated mTOR (P-mTOR), and Rictor), neural differentiation proteins (β3-Tubulin, MAP2, GFAP) and GAPDH. **f** Quantitative comparison of the expression levels of neurofibromin, P-mTOR, Rictor, β3-Tubulin, MAP2, and GFAP in different groups (data are expressed relative to GAPDH). *P < 0.05 compared with the control and LV-vector groups; ^#^*P* < 0.05 compared with the LV-NF-1+LV-Rictor group.

Fourteen days after induction of differentiation, compared with those in the control and LV-vector groups, the proportions of MAP2-positive and β3-Tubulin-positive cells in the LV-NF-1-treated NSCs increased significantly (Fig. 2a, b, d). As expected, the percentages of MAP2-positive and β3-Tubulin-positive cells significantly decreased after the knockout of *Rictor*, whereas the numbers of MAP2-positive and β3-Tubulin-positive cells in the LV-NF-1+LV-Rictor-treated NSCs were significantly superior to those in the LV-Rictor-treated NSCs but were inferior to those in the LV-NF-1-treated NSCs. Similar results for NeuN-positive cells and expression were observed (Supplementary Fig. 2a–d). In contrast, in comparison with that of the control and LV-vector-treated NSCs, GFAP expression was significantly downregulated in both the LV-NF-1-treated NSCs and the LV-NF-1+Rictor-treated NSCs but significantly upregulated in the LV-Rictor-treated NSCs (Fig. 2c, d). To further determine the differentiation of NSCs and the activation of the mTORC2 pathway, we analyzed the expression of relevant proteins by Western blotting. The mTORC2 pathway was enhanced after the knockout of *NF-1*, and the expression levels of proteins related to neural differentiation were similar to those shown in immunofluorescence staining. Specifically, after the knockout of *NF-1*, the expression of phosphorylated mTOR (P-mTOR), Rictor, MAP2, and β3-Tubulin was upregulated. Conversely, after *Rictor* knockout, the mTORC2 pathway was blocked, and the expression levels of P-mTOR, Rictor, MAP2, and β3-Tubulin decreased significantly. Moreover, after coknockout of *NF-1* and *Rictor*, the mTORC2 pathway was blocked, but the expression levels of MAP2 and β3-Tubulin were higher than those after *Rictor* knockout and lower than those after *NF-1* knockout (Fig. 2e, f). These results indicated that knockout of *NF-1* promotes neuronal differentiation and inhibits the astroglial differentiation of NSCs by enhancing the mTORC2/Rictor pathway, but other pathways may be involved.

Furthermore, the direct interaction between NF-1 and Rictor was confirmed by coimmunoprecipitation (Co-IP) experiments. An antibody specific for NF-1 or Rictor was used to pull down its interacting protein, and the presence of NF-1 or Rictor in this complex was determined using Western blotting, with IgG as a control. As expected, Rictor was significantly enriched in the NF-1 protein complex compared to that of either IgG control (Supplementary Fig. 3a), while NF-1 was significantly enriched in the Rictor protein complex compared to that of either IgG control (Supplementary Fig. 3b). Collectively, these results suggest that Rictor is a potential cofactor of NF-1 that regulates the fate of neural stem cells in the treatment of SCI.

The in vitro results prove that the differences in antiapoptotic and neuronal differentiation abilities between the untreated and LV-vector-treated NSCs are not significant. Many studies have confirmed the efficacy of transplanted NSCs in SCI. In the in vivo experiments, we only statistically compared the SCI+LV-vector NSCs, SCI+LV-NF-1 NSCs, SCI+LV-Rictor NSCs, and SCI+LV-NF-1+LV-Rictor NSCs.

### Transplantation of *NF-1* knockout NSCs enhanced neurological recovery after SCI

**Fig. 3:**
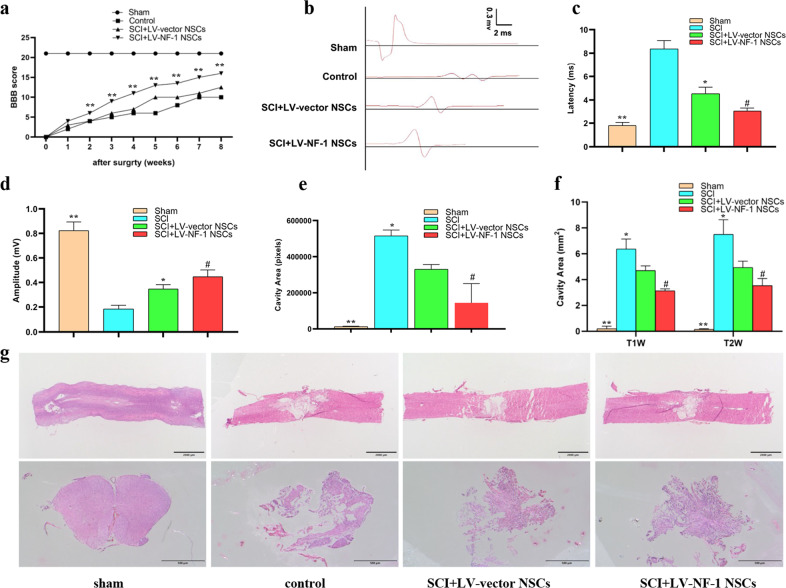
Transplantation of *NF-1* knockout neural stem cells (NSCs) enhanced neurological recovery and reduced lesion volume in rats after spinal cord injury. **a** Basso, Beattie, and Bresnahan (BBB) scores of different groups at different time points. **b**–**d** Comparisons of SCEP latency and amplitude among groups. **e** Quantitative comparisons of the cavity volume from the sagittal section with HE staining among groups. **f** Quantitative comparisons of the cavity volume from T1- and T2-weighted MRI images. **g** Hemotoxylin-eosin (HE) staining of spinal cord sections from different groups (upper panels: sagittal sections, scale bar = 2000 µm; lower panels: transverse sections, scale bar = 500 µm). *P < 0.05 compared with the control group; ^#^*P* < 0.05 compared with the SCI+LV-vector group; ^**^P < 0.05 compared with the other groups.

First, the BBB scores were evaluated weekly after SCI. All rats subjected to SCI had a score of zero on the first day after the injury, which indicates the success of SCI. All injured rats achieved higher BBB scores from the 1st week to the 8th week, and the rats transplanted with the LV-NF-1-treated NSCs achieved notably better BBB scores than those in the other injured groups in each evaluation from the 2nd week, followed by those in the SCI+LV-NF-1+LV-Rictor NSCs, SCI+LV-vector NSCs, SCI+LV-Rictor NSCs and control groups (Fig. 3a). Next, the weight-supported stepping of rats was evaluated at the 8th week after SCI. The rats without SCI could support their weights by hindlimbs easily and walk normally (Supplementary Fig. 4a). The rats in the SCI+LV-NF-1 NSC group could stand up and walk slowly using their hindlimbs, while the rats in the LV-vector NSC-treated group could hardly stand up and could not walk. However, the rats in the control group could not stand up on their hindlimbs. Furthermore, electrophysiological analysis was conducted to assess whether locomotor recovery was correlated with SCEP responses. Compared with those in the sham group, the SCEPs in all SCI groups were weak at the end of the 8 weeks after SCI. However, the SCEP of the LV-NF-1 NSC-treated rats with SCI was stronger than that of the SCI+LV-vector NSC and control groups, expressed as shorter latency and higher amplitude (Fig. 3b–d). These results indicate that the transplantation of NSCs with *NF-1* knockout facilitates the recovery of locomotor function in rats with SCI.

### Transplanted *NF-1* knockout NSCs reduced the lesion volume of SCI

The size of the SCI was assessed by MRI and HE staining at 8 weeks post-operatively. Compared with those of the SCI+LV-vector NSC group, the cavity areas in both the sagittal and transverse spinal cord tissue sections of the SCI+LV-NF-1 NSC group were significantly smaller (Fig. 3e, g). MRI showed similar results, as the lesion area of SCI reflected on the MRI of the SCI+LV-NF-1 NSC group was significantly smaller than that of the SCI+LV-vector NSC group (Fig. 3f).

### Transplantation of NSCs with *NF-1* knockout alleviated apoptosis and enhanced survival of residual neurons

**Fig. 4:**
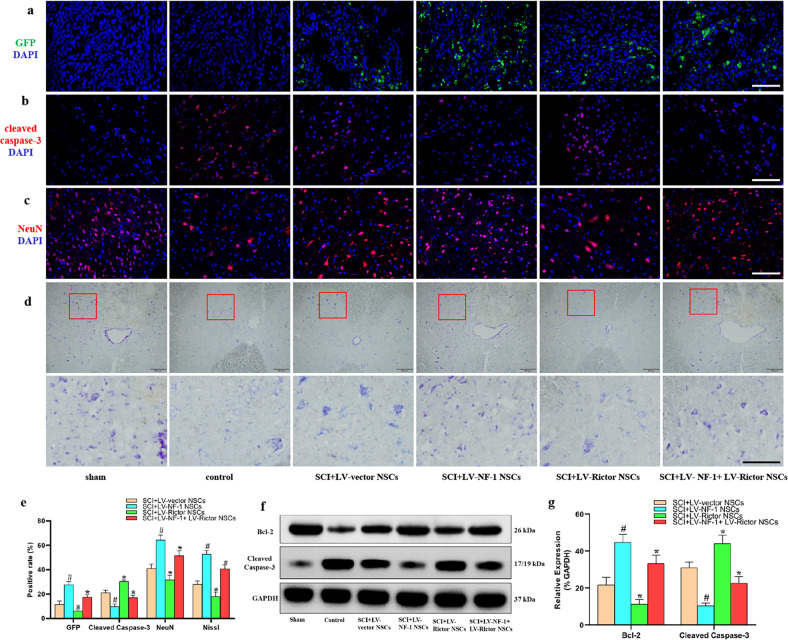
Transplantation of neural stem cells (NSCs) with *NF-1* knockout alleviated apoptosis and enhanced the survival of residual neurons. **a** Survival of transplanted NSCs. Green fluorescent protein (GFP)-positive cells indicate transplanted NSCs (scale bar = 100 µm). **b** Apoptotic cells (cleaved caspase-3 positive) in the epicenter (scale bar = 100 µm). **c** Survival of residual neurons (NeuN-positive) at 5 mm rostral to the epicenter (scale bar = 100 µm). **d** Survival of residual neurons (Nissl positive) at 5 mm rostral to the epicenter (upper panels: lower magnification; lower panels: higher magnification; scale bar = 500 µm). **e** Quantitative comparisons (positive rates) of GFP-, cleaved caspase-3-, NeuN-, and Nissl-positive cells in different groups. **f** Western blotting of apoptosis-related proteins (Bcl-2, cleaved caspase-3, and GAPDH). **g** Quantitative comparisons of the expression of Bcl-2 and cleaved caspase-3 in different groups (data are expressed relative to GAPDH). *P < 0.05 compared with the SCI+LV-vector NSC group; ^#^*P* < 0.05 compared with the SCI+LV-NF-1+LV-Rictor NSC group.

Eight weeks after SCI, GFP was first used as a marker to directly detect the grafted NSCs. Compared with that of the SCI+LV-vector NSC group, the proportion of GFP-positive cells significantly increased in the SCI+LV-NF-1 NSC group but decreased in the SCI+LV-Rictor NSC group. After coknockout of *NF-1* and *Rictor*, however, the proportion of GFP-positive cells was greater than that in the SCI+LV-vector NSC group but lower than that in the SCI+LV-NF-1 NSC group. (Fig. 4a)

Furthermore, the effects of grafted cells on surrounding cells were analyzed. Pronounced cell apoptosis with positive cleaved caspase-3 was observed in the epicenter of SCI. Transplantation of NSCs alleviated cell apoptosis compared with that in the control group. The LV-NF-1-treated NSCs showed the strongest alleviation of cell apoptosis, followed by the NSCs treated with LV-NF-1+LV-Rictor, LV-vector, and LV-Rictor (Fig. 4b). Western blot analysis showed that the expression of cleaved caspase-3 was similar to that found with immunofluorescence staining, while Bcl-2 was expressed in the opposite manner as cleaved caspase-3 (Fig. 4f). The percentage of cleaved caspase-3-positive cells and the relative expression levels of Bcl-2 and cleaved caspase-3 in each group are shown in Fig. 4e, g.

Moreover, the neurons at 5 mm rostral and caudal to the epicenter were examined to determine the effect of transplanted NSCs on the protection of residual neurons. For neuron counting, transverse sections (*n* = 5 per group) of the spinal cord were used to stain neurons with Nissl and NeuN. Five sections at 5 mm rostral and caudal to the epicenter were counted for each rat. The numbers of positively stained cells were counted and averaged per section in a blinded manner. The cells with positive NeuN staining (Fig. 4c, e) and Nissl staining (Fig. 4d, e) were the most abundant in the sham group, followed by the SCI+LV-NF-1 NSC, SCI+LV-NF-1+LV-Rictor NSC, SCI+LV-vector NSC, SCI+LV-Rictor NSC, and control groups.

These results indicate that the transplantation of NSCs after SCI can alleviate cell apoptosis and enhance the survival of surrounding neurons. Furthermore, this function of NSCs can be strengthened after knockout of *NF-1*.

### Knockout of *NF-1* improved neuronal differentiation and enhanced the mTORC2 pathway in transplanted NSCs in vivo

**Fig. 5:**
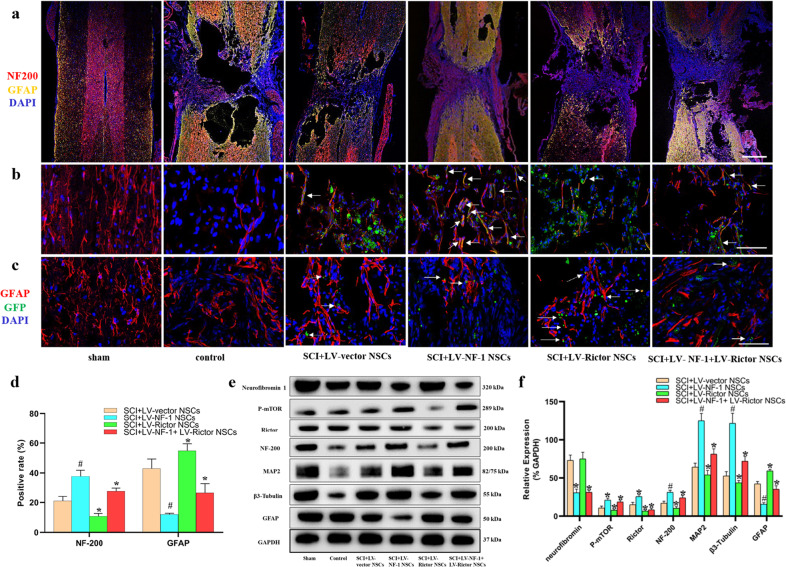
Knockout of *NF-1* improved neuronal differentiation and enhanced the mTORC2 pathway in transplanted neural stem cells (NSCs) in vivo. **a** Tile scan images of spinal cord injury with immunofluorescence staining of neural markers (NF-200 and GFAP) (scale bar = 500 µm). **b**, **c** Immunofluorescence staining of green fluorescent protein (GFP), neurofilament outgrowth marker (NF-200), and astrocyte marker (GFAP) (scale bar = 100 µm). **d** Quantitative comparisons (positive rates) of NF-200- and GFAP-positive cells in different groups. **e** Western blotting of mTORC2 signaling pathway-related proteins (neurofibromin, P-mTOR, and Rictor), neural differentiation proteins (NF-200, MAP2, β3-Tubulin, and GFAP), and GAPDH. **f** Quantitative comparisons of the expression levels of neurofibromin, P-mTOR, Rictor, NF-200, MAP2, β3-Tubulin, and GFAP in different groups (data are expressed relative to GAPDH). *P < 0.05 compared with the SCI+LV-vector NSC group; ^#^*P* < 0.05 compared with the SCI+LV-NF-1+LV-Rictor NSC group.

In addition to the upgraded survival of the LV-NF-1-treated NSCs and residual cells, the neuronal differentiation of the transplanted LV-NF-1-treated NSCs in vivo was also ameliorated. The proportions of cells expressing MAP2 and β3-Tubulin and the neurofilament outgrowth marker (NF-200) in the SCI+LV-NF-1 NSC group were superior to those in the other SCI groups and were reduced stepwise in the SCI+LV-NF-1+LV-Rictor NSC, SCI+LV-vector NSC, SCI+LV-Rictor NSC, and control groups (Fig. 5a, b and Supplementary Fig. 4c, d). GFAP was expressed largely in the control group, followed by the SCI+LV-Rictor NSC group, and was reduced stepwise in the SCI+LV-vector NSC, SCI+LV-NF-1+LV-Rictor NSC, and SCI+LV-NF-1 NSC groups (Fig. 5a, c). Compared with those of the SCI+LV-vector NSC group, the counts of NF-200-, MAP2- and β3-Tubulin-positive cells in the SCI+LV-NF-1 NSC group were 1.8-, 2.1-, and 2.2-fold greater, respectively, while the count of GFAP-positive cells was reduced to 28.3%. Additionally, in the SCI+LV-NF-1+LV-Rictor NSC group, the counts of NF-200-, MAP2-, and β3-Tubulin-positive cells were 1.3-, 1.4-, and 1.4-fold those of the SCI+LV-vector NSC group, respectively, while the count of GFAP-positive cells was reduced to 60% of that of the SCI+LV-vector NSC group. In contrast, grafted NSCs in the SCI+LV-Rictor NSC group differentiated into fewer NF-200-, MAP2- and β3-Tubulin-positive cells (50.7, 45.5, and 48.3% compared with the SCI+LV-vector NSC group, respectively), but the number of GFAP-positive cells was 1.3-fold greater than that in the SCI+LV-vector NSC group (Fig. 5d and Supplementary Fig. 4b).

The Western blot results of MAP2, β3-Tubulin, NF-200, and GFAP were similar to the immunofluorescence staining results. Knockout of *NF-1* increased the expression of MAP2, β3-Tubulin, and NF-200, upregulated the member proteins of the mTORC2 pathway (P-mTOR, Rictor), and reduced the expression of the astrocyte marker GFAP. After *Rictor* knockout, the expression levels of MAP2, β3-Tubulin, and GFAP were contrary to those after *NF-1* knockout. When *NF-1* and *Rictor* were coknocked out, the mTOR2 pathway was blocked, but in comparison with the results after knockout of *Rictor*, the expression of MAP2, β3-Tubulin and NF-200 increased, and the GFAP level decreased. The relative expression levels of relevant proteins in the SCI+LV-vector NSC, SCI+LV-NF-1 NSC, SCI+LV-Rictor NSC, and SCI+LV-NF-1+LV-Rictor NSC groups are shown in Fig. 5e, f.

In accordance with the in vitro results, the in vivo results also indicate that knockout of *NF-1* promotes neuronal differentiation and decreases astroglial differentiation of NSCs by enhancing the mTORC2/Rictor pathway in transplantation of SCI, and other pathways may be involved.

### Transplantation of *NF-1* knockout NSCs facilitated axonal regeneration

**Fig. 6:**
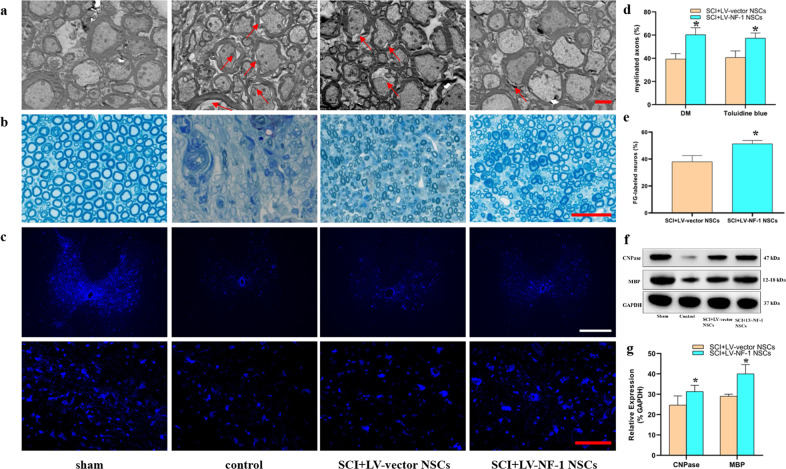
Transplantation of *NF-1* knockout neural stem cells (NSCs) facilitated axonal regeneration. **a** Electron microscopic (EM) visualization of visible demyelinated axons (red arrows) in different groups (scale bar = 2 µm). **b** Sections of toluidine blue staining showed myelinated and demyelinated axons in the different groups (scale bar = 20 µm). **c** Immunohistochemistry showed Fluorogold (FG)-labeled neurons in the T8 segment of the spinal cord, which indicates that neurogenesis can bridge the lesion epicenter (upper panels: lower magnification; lower panels: higher magnification) (scale bar = 100 µm). **d** Quantitative comparisons of proportions of myelinated axons based on DM and toluidine blue staining between the SCI+LV-vector NSC and SCI+LV-NF-1 NSC groups. **e** Quantitative comparisons of FG-labeled neurons at the T8 level between the SCI+LV-vector NSC and SCI+LV-NF-1 NSC groups. **f** Western blotting of CNPase, MBP, and GAPDH. **g** Quantitative comparisons of the expression of CNPase and MBP between the SCI+LV-vector NSC and SCI+LV-NF-1 NSC groups (data are expressed relative to GAPDH). *P < 0.05 compared with the LV-vector group.

Axonal regeneration is another crucial tenet for the treatment of SCI. EM and toluidine blue staining showed that the loss of myelin was apparent in all SCI groups (Fig. 6a, b). However, the percentage of myelinated axons in the SCI+LV-NF-1 NSC group was significantly greater than that in the SCI+LV-vector NSC group (Fig. 6d). The expression levels of CNPase and myelin basic protein (MBP) were measured via Western blots, and the rats treated with LV-NF-1 NSCs expressed markedly higher CNPase and MBP levels than the LV-vector NSC-treated rats (Fig. 6f, g).

Furthermore, the neural circuit plasticity of remyelinated axons was assessed via retrograde tracing using FG. In the sham group, almost all neurons in the ventricles at the T8 level were labeled by FG. In contrast, a significantly smaller number of neurons at the T8 level were FG-positive in all the SCI groups, especially in the control group. Compared with that in the SCI+LV-vector NSC group, the number of FG-positive neurons in the SCI+LV-NF-1 NSC group was significantly higher (Fig. 6c, e). These results indicate that the transplantation of NSCs with *NF-1* knockout facilitates axonal regeneration and that regenerative axons also contribute to axonal plasticity.

## Discussion

The limited survival and neuronal differentiation rates of transplanted NSCs are the major hurdles for neural regeneration and functional recovery after SCI^23^. In this study, we first demonstrated that the knockout of *NF-1* improved the antiapoptotic and neuronal differentiation abilities of NSCs via the enhancement of the mTORC2 signaling pathway in vitro and in vivo, and the transplantation of NSCs with *NF-1* knockout increased tissue repair and neurological recovery in rats subjected to SCI. In contrast, knockout of *Rictor* decreased NSC survival and neuronal differentiation. In comparison with the *Rictor* knockout, coknockout of *NF-1* and *Rictor* could partly strengthen the survival and neuronal differentiation of NSCs.

Accumulated evidence has shown the poor vitality and low neuronal differentiation efficiency of exogenous NSCs in the injured site of SCI due to primary damage and secondary injury^24^. Consequently, increasing the vitality and neuronal differentiation efficiency of transplanted NSCs is crucial. An increasing number of studies have revealed that the differentiation of NSCs can be potentiated by regulating intercellular mechanisms^19,25^. Previous studies have demonstrated that *NF-1* regulates the cell proliferation and astroglial or oligodendroglial differentiation of NSCs in neurogenesis, but whether *NF-1* modulates the neuronal differentiation of NSCs in the treatment of SCI remains unexplored. In the present study, decreased apoptosis was first identified in NSCs after *NF-1 k*nockout. Studies have revealed the regulation of *NF-1* in NSC proliferation and multilineage differentiation through distinct RAS effector pathways^12^. Moreover, *NF-1* inactivation of NSCs resulted in increased glial lineage proliferation and abnormal neuronal differentiation in the developing brain by both cAMP- and Ras-dependent mechanisms^26^. Thus, we explored the molecular mechanism underlying the function of *NF-1* in NSCs in this study.

Recently, the mTORC2 signaling pathway was proven to mediate the survival, proliferation, and neurogenesis of cortical progenitor cells^27^. It was reported that mTORC2/Rictor could promote cell survival by phosphorylating AKT and controlling its activity^28^ and decrease cell apoptosis by stabilizing the antiapoptotic protein through suppression of its degradation. In our study, the loss of *NF-1* promoted the survival and neuronal differentiation of NSCs in SCI via the hyperactivation of the mTORC2 pathway. Furthermore, the inhibition of the mTORC2 signaling pathway substantially attenuated the survival and neuronal differentiation of *NF-1*-silenced NSCs. Consistent with previous studies, our results suggested the vital role of the mTORC2 signaling pathway in the regulation of cell apoptosis and neuronal differentiation in *NF-1*-silenced NSCs. In addition, the immune-modulating function of NSCs was verified^29^, and NSCs with the enhancement of the mTORC2 pathway not only significantly attenuated the acute inflammatory response and cell death after SCI but also markedly increased the shift in macrophages around the lesion from the M1 to the M2 phenotype^18^. The loss of *NF-1* activated the mTORC2 pathway, induced NSCs to mainly differentiate into neurons, and promoted tissue repair and neurological recovery in rats with SCI. This result provided strong evidence that mTORC2 is a molecular therapeutic target to optimize the transplantation of NSCs after SCI. Interestingly, after the simultaneous knockout of *NF-1* and *Rictor* in NSCs, the antiapoptotic and neuronal differentiation effects were inferior to those of *NF-1* knockout but superior to those of the knockout of *Rictor* alone, suggesting that the loss of *NF-1* might also regulate the fate of NSCs via other mechanisms. Therefore, our findings complemented the theoretical gap of *NF-1* in the neuronal differentiation of NSCs.

The improvements in the antiapoptotic effects and neuronal differentiation of NSCs facilitated locomotor function and tissue repair in rats with SCI. Significantly higher BBB scores and SCEP amplitudes were detected in the rats with transplanted *NF-1* knockout NSCs, suggesting that the transplanted *NF-1* knockout NSC-differentiated neurons were also able to form electrophysiologically active neuronal relays and extend numerous axons for long distances both caudally and rostrally from the injury, improving the functional status of motor and sensory axonal conductions^30^. The main mechanisms of NSC transplantation in SCI include reducing the glial scar by modulating astrocytes and enhancing neuronal differentiation and oligodendrocyte differentiation^31^. SCI lesions are formed by three tissue compartments, fibrotic scars, astroglial scars, and viable neural tissue surrounding the border of the astroglial scar^32^. On the one hand, the astroglial scar limits the lesion; on the other hand, the existence of the astroglial scar obstructs the connection of rostral and caudal neurons^33^. In the present study, NSCs with *NF-1* knockout differentiated into neurons, and neurofilament outgrowth was significantly enhanced. In contrast, infiltrative astrocytes and glial scar formation were markedly decreased. These results provide robust evidence supporting the heightened neuronal differentiation and regulation of glial scar formation by *NF-1* knockout NSCs in the treatment of rats with SCI. The differentiation of neurons and neurofilament outgrowth could directly replace the lost and injured inborn neurons^31^. However, the reestablishment of neural connections that rely on axonal regeneration is another crucial component of traumatic SCI^30,34^ because axonal dieback emerges at early stages, and axonal regeneration is severely inhibited after SCI^35^. Mounting evidence indicates that the ideal therapy to induce axonal regeneration in SCI should reduce scarring and growth-inhibitory factors and reactivate the growth potential of axons^36^. However, the function of reactive astrocytes in secreting inhibitory molecules and blocking axonal regeneration has been proven^37^. In this study, myelin regeneration based on electron microscopy analysis and the results of retrograde tracing by fluorogold indicated that axonal regeneration was significantly increased due to the transplantation of *NF-1* knockout NSCs. This phenomenon may be attributed to the enhanced regulation of scar formation, reduction in infiltrative astrocytes, and neuromodulation of *NF-1* knockout NSCs.

In addition to direct damage to neural cells in the epicenter, apoptosis of residual cells around the epicenter also influences neurological recovery^38^, and injury-induced apoptosis can be observed in all types of cells after SCI^39^. The apoptosis of cells is considered the continuation of cellular destruction derived from secondary injury following SCI and may be the main mechanism of progressive expansion of the lesion area^40^. The transplantation of stem cells could release several neurotrophic factors to ameliorate the harsh postinjury microenvironment and facilitate the survival of residual neurons^41,42^. Apoptosis is a caspase‐dependent cell death modality^43^. The decreased cleaved caspase-3-positive cells in the lesion and the increased residual neurons surrounding the lesion in the *NF-1* knockout NSC-treated rats with SCI indicated that the transplantation of NSCs in rats with SCI could moderate the apoptosis of residual cells, and the ability of *NF-1* knockout NSCs was enhanced significantly. The enhanced ability of *NF-1* knockout NSCs to moderate the apoptosis of residual cells may be attributed to the enhanced cell vitality owing to the enhanced mTORC2 pathway.

The function of *NF-1* in apoptosis and neuronal differentiation of NSCs and the preliminary mechanism were first verified in our study. Further investigation with RNA-seq and Ingenuity Pathway Analysis (IPA) is needed to determine the exact molecular mechanism between *NF-1* silencing and the effect on cellular functions.

In summary, we have demonstrated that the knockout of *NF-1* improved the apoptosis and neuronal differentiation of NSCs via the enhancement of the mTORC2 signaling pathway in vitro, and the transplantation of *NF-1* knockout NSCs is sufficient to promote the tissue repair and locomotor recovery of rats with SCI by enhancing the survival, apoptosis and neuronal differentiation abilities of NSCs. These findings reveal a prominent role of *NF-1* in NSC biology, which is an invaluable step forward in enhancing the benefit of NSC-mediated regenerative cell therapy for SCI and demonstrates that transplantation of *NF-1* knockout NSCs is a promising strategy for SCI.

## Supplementary information

Supplemental Figures
