# Supplementary material for: Transplanting neurofibromatosis-1 gene knockout neural stem cells improve functional recovery in rats with spinal cord injury by enhancing the mTORC2 pathway
Source: Exp Mol Med. 2022 Oct 14;54(10):1766–77. doi: 10.1038/s12276-022-00850-9 (PMC9636387; doi:10.1038/s12276-022-00850-9)
Supplement: Supplementary file 1 — Supplemental Figures [file 12276_2022_850_MOESM1_ESM.pdf]

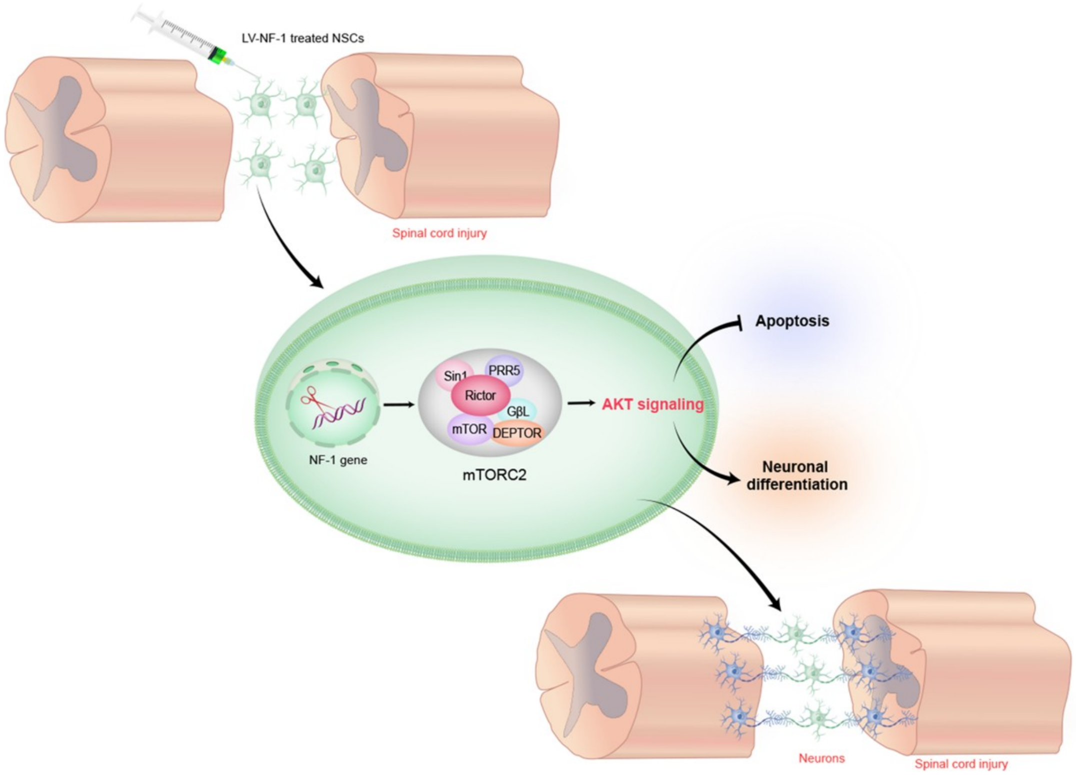

Supplementary Fig. 1 Graphical abstract of this study.

LV-NF-1 indicates neurofibromatosis-1 gene (*NF-1*) sgRNA-CAS9 lentivirus, NSCs indicates neural stem cells.

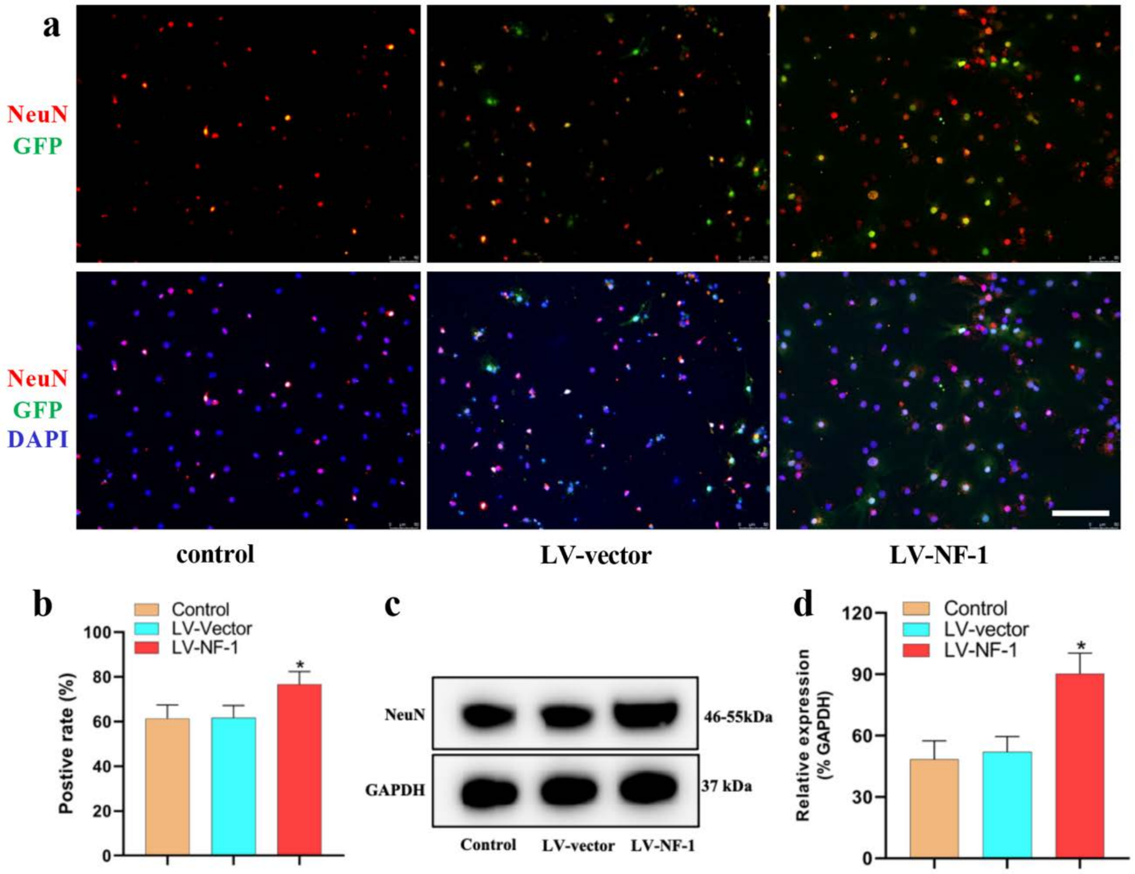

Supplementary Fig. 2 Knockout of *NF-1* promoted neuronal differentiation of neural stem cells (NSCs).

a. Immunofluorescent staining of NeuN and green fluorescent protein (GFP), (scale bar=100 μm). **b.** Quantitative comparison (positive rate) of NeuN-positive cells in different groups. **c.** Western blotting of NeuN and glyceraldehyde-3-phosphate dehydrogenase (GAPDH). **d.** Quantitative comparison of expression of NeuN in different groups (data was expressed as relative to GAPDH).

* P<0.05, compared with Control and LV-vector groups.

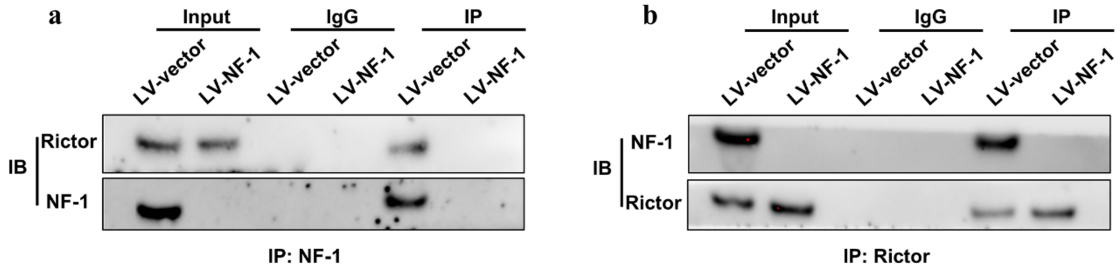

Supplementary Fig. 3 Co-immunoprecipitation (Co-IP) of NF-1 and Rictor proteins in LV-vector- and LV-NF-1-treated neural stem cells (NSCs).

Direct interaction between NF-1 and Rictor was confirmed by Co-IP experiments. Antibody specific for NF-1 or Rictor was used to pull down its interacting protein and the presence of NF-1 or Rictor in this complex were determined using Western blotting, with IgG as control. **a.** Rictor was significantly enriched in the NF-1 protein complex compared to either IgG control. **b.** NF-1 was significantly enriched in the Rictor protein complex compared to either IgG control.

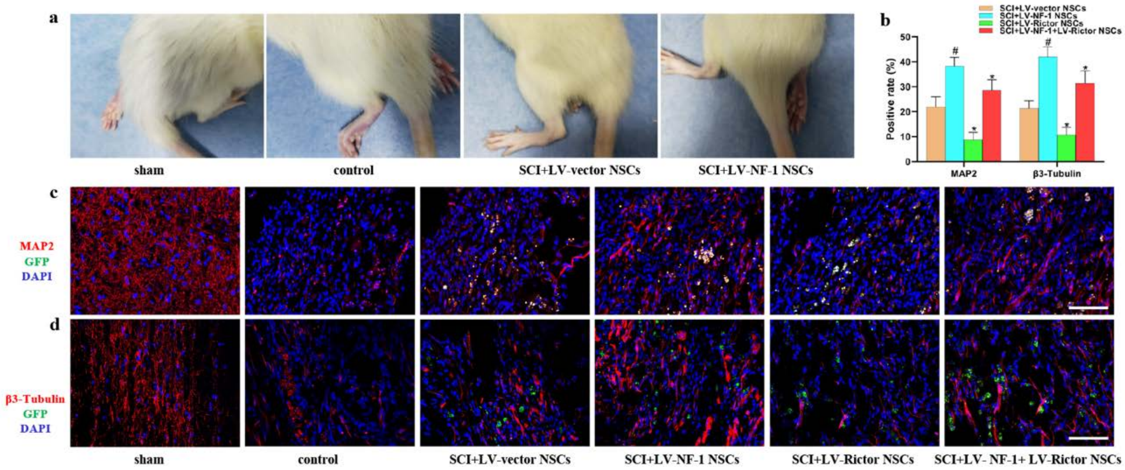

Supplementary Fig. 4 Transplantation of *NF-1* knockout neural stem cells (NSCs) enhanced neurological recovery and neuronal differentiation of rats after spinal cord injury (SCI).

a. Weight-supported stepping of rats at 8 weeks after SCI of different groups. Rats without SCI can support their weight by hindlimbs easily and walk normally; rats in the Control groups cannot stand up using hindlimbs, while rats in the LV-vector NSCs group can stand up hardly but not walk; rats in the SCI+LV-NF-1 NSCs group can stand up and walk slowly using hindlimbs. **b**. Quantitative comparisons (positive rates) of MAP2- and β3-Tubulin-positive cells in different groups. **c-d**. Immunofluorescent staining of green fluorescent protein (GFP) and neuronal markers (β3-Tubulin and MAP2), scale bar=100 μm.

* P<0.05 compared with SCI+LV-vector NSCs group; # P<0.05 compared with SCI+LV-NF-1+LV-Rictor NSCs group.
